# Gynecologic oncology patients' satisfaction and symptom severity during palliative chemotherapy

**DOI:** 10.1186/1477-7525-4-84

**Published:** 2006-10-30

**Authors:** Vivian E von Gruenigen, Jessica R Hutchins, Anne Marie Reidy, Heidi E Gibbons, Barbara J Daly, Elisa M Eldermire, Nancy L Fusco

**Affiliations:** 1 University Hospitals Case Medical Center, Cleveland, Ohio 44106, USA; 2 Case Western Reserve University, 11100 Euclid Avenue, Cleveland, Ohio 44106, USA

## Abstract

**Background:**

Research on quality and satisfaction with care during palliative chemotherapy in oncology patients has been limited. The objective was to assess the association between patient's satisfaction with care and symptom severity and to evaluate test-retest of a satisfaction survey in this study population.

**Methods:**

A prospective cohort of patients with recurrent gynecologic malignancies receiving chemotherapy were enrolled after a diagnosis of recurrent cancer. Patients completed the Quality of End-of-Life care and satisfaction with treatment scale (QUEST) once upon enrollment in an outpatient setting and again a week later. Patients also completed the Mini-Mental Status Exam, the Hospital Anxiety/Depression Scale, a symptom severity scale and a demographic survey. Student's t-test, correlation statistics and percent agreement were used for analysis.

**Results:**

Data from 39 patients were analyzed. Mean (SD) quality of care summary score was 41.95 (2.75) for physicians and 42.23 (5.42) for nurses (maximum score was 45; p = 0.76 for difference in score between providers). Mean (SD) satisfaction of care summary score was 29.03 (1.92) for physicians and 29.28 (1.70) for nurses (maximum score was 30; p = 0.49 for difference between providers). Test-retest for 33 patients who completed both QUEST surveys had high percent agreement (74–100%), with the exception of the question regarding the provider arriving late (45 and 53%). There was no correlation between quality and satisfaction of care and symptom severity. Weakness was the most common symptom reported. Symptom severity correlated with depression (r = 0.577 p < 0.01). There was a trend towards a larger proportion of patients reporting pain who had three or more prior chemotherapy regimens (p = 0.075). Prior number of chemotherapy regimens or time since diagnosis was not correlated with symptom severity score. Anxiety and depression were correlated with each other (r = 0.711, p < 0.01). There was no difference in symptom severity score at enrollment between those patients who have since died (n = 19) versus those who are still alive.

**Conclusion:**

The QUEST Survey has test-retest reliability when used as a written instrument in an outpatient setting. However, there was no correlation between this measure and symptom severity. Patient evaluation of care may be more closely related to the interpersonal aspects of the health care provider relationship than it is to physical symptoms.

## Background

Understanding patient perceptions of technical and interpersonal care they receive and satisfaction with that care is essential. Assessments of quality and satisfaction of care in oncology have focused on patients' satisfaction with physicians or the health care system [1,2]. Research on satisfaction during palliative care and care at the end of life (EoL) of cancer patients has been limited [3]. Global measures of quality and satisfaction with care are not completely revealing, because they do not indicate on which issues, such as symptom management, the provider should focus on improving [4].

Patients' satisfaction with care may be significantly affected by their symptoms and the physician's response to these symptoms, particularly during the advanced stages of cancer. Gynecologic cancer symptoms are multi-factorial in character as the primary cancer frequently metastasizes to other pelvic and abdominal organs. Women with ovarian cancer present with a constellation of symptoms including back pain, fatigue, abdominal pain and urinary symptoms [5]. Ferrell et al assessed patients with ovarian cancer post-diagnosis; pain, fatigue and gastrointestinal effects were the most problematic [6]. Sun et al revealed that fatigue was a significant problem including higher levels of distress in ovarian cancer patients with recurrent disease [7]. However, the extent to which satisfaction with care is related to perceptions of concern and efforts by providers or to underlying patient mood state, not just to symptoms, is not known [8].

The purpose of this study was to examine the relationship between patients' perception of quality and satisfaction with care and symptom severity during palliative chemotherapy for recurrent gynecologic malignancies. In 2004, the National Cancer Institute declared the importance of improving symptom management for cancer patients [9]. However, there is currently no information on the link between this population's symptoms, anxiety, depression and perception of the quality of cancer care directly influenced by clinicians. In addition, we wanted to evaluate the test-retest properties of the Quality of End-of-Life care and satisfaction with treatment scale (QUEST) Survey in this study population.

## Methods

Prospective patients with gynecologic malignancies receiving chemotherapy were enrolled after a diagnosis of recurrent cancer in this IRB approved study. Patients were seen in the oncology clinic office by their treating gynecologic oncologist and chemotherapy nurse specialist and informed consent was obtained for participation in this study. Patients received a variety of chemotherapy agents depending on prior treatment and patient/physician preferences. Eligibility criteria included age of 18 or greater and a mini-mental status exam score of 12 or higher. Patients completed the QUEST survey regarding quality of care and satisfaction with care received from both their physicians and nurses. The survey was completed once upon enrollment in an outpatient setting and again a week later. Patient responses were placed in a sealed envelope and they were assured that their individual responses would not be revealed to their treating physician or nurse. Patients also completed the Mini-Mental Status Exam, the Hospital Anxiety/Depression, a symptom severity scale and a demographic survey. Patient charts were reviewed to obtain demographic and clinical variables.

### Measures

The QUEST Survey contains fifteen items categorized into two sub-scales in which patient's rate the quality of the care they have received from their physicians and nurses separately, and their satisfaction with care. Quality (nine questions) was rated using a 5-point Likert scale to assess how often particular behaviors or styles of care were true of their health care providers. Ratings ranged from never to always. Similarly, satisfaction (six questions) was rated using a 5-point Likert scale ranging from "very dissatisfied" to "very satisfied". Items for each scale were summated to obtain an overall score for both quality and satisfaction with care [3].

Folstein et al developed a simplified, scored form to evaluate mental state. The Mini-mental status exam (MMSE) includes eleven questions (maximum score of 30), requires 5–10 minutes to administer and is practical for use serially and routinely. The MMS concentrates on the cognitive aspects of mental functions and has documented validity and reliability [10]. This evaluation tool was used to screen patients at enrollment for any mental deficiencies. In addition this tool has not been used before in this patient population. The Hospital Anxiety and Depression Scale (HAD) has been established as a convenient self-rating screening instrument for anxiety and depression [11,12]. The survey consists of fourteen multiple choice items that are scored on a scale of 0 to 3 and questions are categorized as measuring anxiety versus depression accordingly. A score of 8 or higher on either scale indicates the possibility that the patient may have an anxiety or depression disorder and should be evaluated further. Previous research in this population has indicated increased levels of anxiety/depression [7].

A symptom severity scale adapted from that of Mercadante et al was used to analyze frequency and severity of common gynecologic cancer symptoms [13]. Symptoms included pain, shortness of breath, nausea/vomiting, weakness and drowsiness and were included in a standard form and rated for severity (absent 0, mild 1, moderate 2, severe 3). A brief demographic survey regarding religious affiliation and educational level was completed by patients.

### Statistical analysis

Patient demographics and clinical characteristics were summarized using descriptive statistics. Student's t-test was used to compare QUEST scores between physicians (MD) and nurses (RN) in all patients and correlation analysis was done between the HADS, symptom severity scale and QUEST surveys in order to determine if increased symptoms were association with increased depression or anxiety and decreased satisfaction score. Patients completing both QUEST surveys (n = 33) were used to compare scores between the initial survey and a second survey administered one week later. Percent agreement, correlations, and paired t-tests were used to compare scores for patients completing the survey at both time points. Symptom severity was analyzed in only ovarian cancer patients by correlation analysis and chi-square statistics. The other gynecologic malignancies were not included in this analysis as the number of cases were small and interpretation may not be applicable to other cancer types.

## Results

Forty-four patients were approached regarding the study and 41 enrolled in this prospective study from September 2003 – March 2006. Two patients after enrollment refused to continue due to time constraints. Patient demographics and clinical characteristics of 39 patients with complete data are summarized in Table 1. The majority of patients were married, Caucasian and had some college or higher education (57%). Gynecologic cancers included 79% ovarian cancer, 18% endometrial cancer and 2.5% vaginal cancer. Mini-mental status exam scores were high for all patients (range 27–30) and no patients were excluded based on this exam.

**Table 1:** Patient demographic and clinical characteristics (n = 39)

Age, mean (SD)	60.33 (10.1) years
Marital status	
Married	27 (69%)
Widowed	6 (15%)
Single	4 (10%)
Unknown	2 (5%)
Race	
Caucasian	32 (82%)
African-American	4 (10%)
Other	3 (7%)
Education	
< HS grad	2 (5%)
HS grad	15 (38%)
Some college	12 (31%)
College grad or higher	10 (26%)
Cancer type	
Ovarian/peritoneal	31 (79%)
Endometrial	7 (18%)
Vaginal	1 (3%)
Time since original diagnosis, mean (SD)	9.6 (12.7) months
Number of prior chemotherapy regimens, median (range)	2 (1–8)

Mean scores for both physicians and nurses regarding quality of care and satisfaction with care received were high (Table 2). There were no differences in scores between providers (MD versus RN). Thirty-three patients completed both QUEST surveys. Mean scores on surveys were compared and there were no differences between scores on the first and second survey. In addition, correlation coefficients were high (Table 3). Percent agreement between surveys for individual questions was calculated. With the exception of question #2 (provider arriving late for appointment), agreement between answers obtained on both occasions was high (Table 4).

**Table 2:** Mean (SD) scores for QUEST survey (n = 39)

	**MD**	**RN**	P value *
**Quality of care**	41.95 (2.75)	42.23 (5.42)	0.76
**Satisfaction with care**	29.03 (1.92)	29.28 (1.70)	0.49

* Student's t-test used for comparison between providers

**Table 3:** Comparison of QUEST surveys in patients completing both initial and second survey (n = 33)

	**Initial survey**	**Second survey**	P value **	**Correlation**	**p value**
**Quality of care, MD**	41.77 (0.50) *	41.9 (0.54)	0.625	0.87	< 0.01
**Quality of care, RN**	41.82 (1.01)	42.42 (0.49)	0.551	0.82	< 0.01
**Satisfaction with care, MD**	28.97 (0.35)	28.24 (0.45)	0.063	0.65	< 0.01
**Satisfaction with care, RN**	29.18 (0.32)	28.73 (0.38)	0.17	0.57	< 0.01

* Mean ± SEM

** paired t-test used for comparison of scores

**Table 4:** Percent agreement for the QUEST survey in patients completing both initial and second survey (n = 33)

	**MD**	**RN**
**Quality of care**		
Spent enough time	90%	79%
Arrived late to see you	***45%***	***53%***
Been hard to reach in time of need	84%	76%
Seemed distracted by other things when you talk	87%	84%
Been willing to take time to listen	84%	84%
Treated you more as a disease than as a person	97%	97%
Showed personal concern about you	84%	88%
Ignored your feelings	97%	94%
Responded quickly in time of need	80%	81%
**Satisfaction with care**		
Bedside manner	84%	82%
Common courtesy	87%	85%
Way of talking to you	74%	82%
Clinical and technical skills in treating you	84%	79%
Concern for you as an individual	87%	85%
Overall level of satisfaction with care provided over past 2 months	90%	85%

Thirteen patients (33%) had an anxiety score greater than 8 and 5 patients (13%) had a depression score of 8 or higher. Anxiety and depression were highly correlated with each other (r = 0.711, p < 0.01). Patients with increased scores were referred to a psychologist within our department for possible treatment.

Symptom severity data, available only for ovarian cancer patients (n = 31), were used to examine patterns and relationships with satisfaction (Table 5). There was no correlation between quality of care and satisfaction scores on the QUEST with symptom severity (r = 0.085 and r = 0.009 respectively). Weakness was the most common symptom reported, and 10 patients (32%) reported no symptoms whatsoever. Symptom severity was correlated with depression (r = 0.577, p = 0.001), but not anxiety. Prior number of chemotherapy regimens or time since diagnosis was not correlated with overall symptom severity score. When patients were stratified based on number of prior chemotherapy regimens, there was a trend towards more frequent reports of pain in patients who had undergone more chemotherapy regimens. Five out of the nine patients (55%) who had undergone three or more chemotherapy regimens reported pain, compared to four out of the 18 patients (22%) with one or two regimens (p = 0.075).

**Table 5:** Frequency and severity of symptoms reported by ovarian cancer patients (n = 31)

**Symptom**	**No (%)**
Weakness	14 (45%)
Mild	11
Moderate	3
Pain	9 (29%)
Mild	9
Drowsiness	9 (29%)
Mild	8
Moderate	1
Shortness of Breath	6 (19%)
Mild	5
Moderate	1
Nausea/Vomiting	6 (19%)
Mild	6

Nineteen patients have died since the study began, all of whom were enrolled during the years 2003–2004. There was no difference in symptom severity score at enrollment between patients who have died versus those still alive.

## Discussion

In this prospective, observational study there was no correlation between perceptions of quality and satisfaction with care and symptom severity. Clinical variables, such as prior number of chemotherapy regimens or time since diagnosis, also were not related to the symptom severity score. There was a trend towards a larger proportion of patients who had multiple prior chemotherapy regimens reporting pain. Weakness was the most common symptom reported. Anxiety and depression were correlated with each other and symptom severity was correlated with depression. We also found the QUEST Survey to have test-retest reliability when used as a written instrument in an outpatient setting.

Quality cancer care includes provision of the most effective curative therapies, as well as excellent symptom management and sensitive end-of-life care. Symptom management, the core of palliative care, is an integral part of cancer care throughout the disease trajectory, while "end-of-life" care usually refers to care during the terminal phase or last few weeks or months of life. There is no objective dividing line between palliative care and end-of-life care and we use the terms interchangeably [6] in this paper to differentiate them from curative aspects of care. In that the majority of patients in our sample had ovarian cancer, which typically recurs after an initial remission, goals of palliative therapy include both prolonging survival as well as maintaining or improving quality of life. Weakness and fatigue are problematic in women with gynecologic cancer, especially ovarian cancer patients who receive multiple chemotherapy regimens. In an interesting research design, Ferrell et al. abstracted data from "Conversations!", a newsletter for those with ovarian cancer in which patients publish their commentary [6]. Data were abstracted from personal stationery, greeting cards, and e-mail. In the pre-diagnostic complaints, fatigue was secondary only to bloating/abdominal swelling. Sun et al. assessed 70 patients with ovarian cancer undergoing chemotherapy for primary or recurrent disease [7]. While nausea and vomiting were the most problematic, fatigue also was a significant problem and higher levels of distress were associated with recurrent disease. These data suggest that there may be a predictable progression of symptoms from the initial abdominal discomfort to progressive weakness and fatigue. Thus it may be helpful for clinicians to specifically assess for these symptoms and prepare patients for their occurrence.

The QUEST survey focuses on the patient's perception of provider's time, access, and communication. In our study scores were consistently high and there were no differences between nurses and doctors as health care providers. Patients were seen in the oncology clinic office usually by the same gynecologic oncologist and chemotherapy nurse specialist. Patients were assured that their responses to questionnaires would be kept anonymous. It is possible, however, that patients may have provided answers that they felt their health care provider expected to hear and did not feel as if they could express negative feelings. Sulmasey et al revealed differences with this survey between physicians and nurses. However, this instrument may not be sensitive enough to detect variations in clinics where patients receive consistent care from the same attending physicians and nurses [3]. It is also possible that this questionnaire is not sensitive enough to pick up small fluctuations in care.

This study revealed no correlation between satisfaction with care and symptom severity. This may be a function of the limited variance in the quality of satisfaction measures. However, it also may suggest that patient evaluation of care is related more to the interpersonal aspects (trust, caring) of the physician-patient or nurse-patient relationship than it is to physical symptoms. If the patient feels confident in the health care providers and perceives them to be sincerely concerned, even if the symptom management is not completely effective, the patient remains satisfied. This reinforces the importance of providers focusing on interpersonal communication, as well as provision of technically competent care, to improve satisfaction with care.

Weaknesses of the study include the limited sensitivity of the QUEST survey with this population of patients. Because of the multidimensional nature of quality of care, a single measure cannot provide a complete assessment of impact. Recent instruments to evaluate symptom severity and satisfaction with care have been developed and may be more appropriate for use in future studies [14,15]. Other options in quality of life (QOL) measures could include FACIT-Pal for palliative care [16], FACIT-TS-PS for treatment satisfaction [16], and possibly the Missoula-VITAS QOL index designed to measure QOL of patients with advance incurable diseases, weighing each dimension according to patient-reported importance [17]. An additional limitation was the brief measure of fatigue, which was the major symptom in this population. A detailed fatigue measure such as the FACIT-F should be administered to expand on the symptom evaluation for further interventions [16]. Many of the above tools were unavailable at the beginning of this study in 2003. Future directions include an ongoing intervention trial targeting symptom improvement in ovarian cancer patients during palliative chemotherapy.

## Conclusion

The QUEST Survey does demonstrate adequate test-retest reliability when used as a written instrument in an outpatient setting with gynecologic oncology patients. High satisfaction and quality of care scores were obtained; however, it may be that a variety of research instruments should be used to evaluate this health care domain. In this pilot investigation of women receiving palliative chemotherapy the most common symptom was weakness. In addition, anxiety and/or depression were observed in over a third of patients in this study population. As patients' cancer progresses despite chemotherapy, they should be frequently assessed and offered interventions for cancer symptoms.

## Competing interests

The author(s) declare that they have no competing interests.

## Authors' contributions

VVG and AMR conceived of the study and design. VVG, HG, JH, EE, NF coordinated the study. VVG and HG were responsible for day to day conduct of the study and analyzed the data. VVG, HG and BD drafted the manuscript; AMR, JH, BD, EE and NF provided critical review. All authors read and approved the final manuscript.
